# The ten “hard” questions in pediatric functional constipation

**DOI:** 10.1186/s13052-024-01623-y

**Published:** 2024-04-08

**Authors:** Flora Fedele, Maria Teresa Fioretti, Elena Scarpato, Massimo Martinelli, Caterina Strisciuglio, Erasmo Miele

**Affiliations:** 1 Department of Translational Medical Science, Section of Pediatrics, University of Naples Federico II, Via Pansini 5, 80131 Naples, Italy; 2 Department of Woman, Child and General and Specialized Surgery, University of Campania “Luigi Vanvitelli”, Naples, Italy

**Keywords:** Chronic constipation, Functional gastrointestinal disorders, Children, Adolescents, Laxatives, Polyethylenglicole

## Abstract

Functional constipation is a common problem in childhood and has a great impact on social, physical, and emotional functioning of affected children and their caregivers. No organic cause of the constipation can be found in approximately 95% of children, defining the “so-called” chronic functional constipation. Its prevalence has been reported to range from 0.7 to 29.6%, with a median of 12%. The diagnosis of functional constipation is exclusively clinical based on the pediatric diagnostic Rome criteria for functional gastrointestinal disorders and does not routinely require laboratory and/or radiological investigations. In case of alarm signs and symptoms that may suggest organic diseases, further investigations can be required. The therapeutic management is based on non-pharmacological and pharmacological approaches. Education, demystification of constipation and reward-based toilet training represent the cornerstones of nonpharmacological management. Disimpaction, maintenance treatment and weaning of medication are all elements of pharmacological treatment. Osmotic laxatives, mainly polyethylene glycol (PEG), are considered the first-choice laxative for both disimpaction and maintenance treatment. The aim of this review is to provide pediatric gastroenterologists with a practical tool to support the clinical and therapeutic management of children and adolescents affected by chronic functional constipation.

## Key points in the management of pediatric functional constipation

1. FC should be diagnosed using Rome IV criteria.

2. FC is the most common functional gastrointestinal disorder in children.

3. Pathophysiology of FC is multifactorial but the stool withholding behavior seems to be the main etiological agent.

4. FC is the most frequent cause of abdominal pain and fecal incontinence in childhood.

5. In the presence of alarm symptoms or in case of failure of conventional treatments, further investigations may be recommended.

6. When education, lifestyle, diet and toilet training are not sufficient in the management of FC, the next step is the pharmacological treatment.

7. Polyethylene glycol (PEG) is the first choice in the treatment of FC in children, both for disimpaction and for maintenance treatment.

8. Maintenance treatment should continue for at least 2 months. After resolution of constipation symptoms, treatment should be decreased gradually.

9. A normal physical activity, a normal fiber and fluid intake is recommended in children with FC.

10. A strict follow-up is highly recommended to avoid persistence of FC symptoms.

## How is functional chronic constipation defined?

Constipation is described as a reduction of normal stool frequency and bowel movements, painful defecation, passage of hard stools, and/or sensation of incomplete evacuation of stools [1]. In 90–95% of cases it is a functional gastrointestinal disorder (FGID), without any underlying systemic cause or anatomical defect [2].

Currently, functional constipation (FC) is defined by the Rome criteria, in the most recent version of the Rome IV criteria (Table 1). A differentiation between toilet-trained and non-toilet-trained children was included in this version. In toilet-trained children, additional criteria such as the presence of episodes of incontinence and a history of heavy stools that may obstruct the toilet are evaluated. Indeed, episodes of fecal incontinence do not apply to children who are not toilet-trained [3, 4].

**Table 1:** ROMA IV criteria for functional constipation

**Must include for 1 month at least two of the following in infants up to 4 years of age:**
- Two or fewer defecations per week;
- History of excessive stool retention; BLUE
- History of painful or hard bowel movements;
- History of large diameter stools; BLUE
- Presence of a large fecal mass in the rectum.
In toilet-trained children, the following additional criteria may be used:
- At least one episode/week of fecal incontinence after the acquisition of toileting skills;
- History of large-diameter stools that may obstruct the toilet.BLUE
**Must include 2 or more of the following occurring at least once per week for a minimum of 1 month with insufficient criteria for a diagnosis of irritable bowel syndrome in children older than 4 years:**
- 2 or fewer defecations in the toilet per week in a child of a developmental age of at least 4 years BLUE
- At least 1 episode of fecal incontinence per week
- History of retentive posturing or excessive volitional stool retention BLUE
- History of painful or hard bowel movements
- Presence of a large fecal mass in the rectum BLUE
- History of large diameter stools that can obstruct the toilet.

Assessment of stool consistency is important in the evaluation of a child’s defecation pattern in the diagnosis of FC [5]. The most commonly used visual stool scale is the Bristol Stool Form Scale, which consists of 7 descriptions of different stool forms accompanied by drawings, ranging from hard stools to watery stools [6]. It is included in the Rome IV questionnaire above 4 years. In young children who are not toilet-trained, its reliability has been debated, and a different visual stool scale was developed for such patients: the Brussels Infant and Toddler Stool Scale [7].

Even though the Rome criteria provide a clear definition of FC, there is no universal definition of refractory constipation in pediatrics. This condition has never been formally defined and standardized; it is generally defined as failure of maximal laxative therapy and/or need for daily rectal stimulation (enemas or suppositories) for > 3 months. In these patients it is necessary to carefully evaluate compliance with treatment and exclude the presence of secondary causes of constipation, before escalating to more invasive treatments, including the new pharmacological agents not yet approved in pediatrics [8, 9].

## What is the incidence?

The heterogeneity of the studies in terms of population sampling, diagnostic criteria, ethnicity and background of the participants does not allow to determine the true prevalence of FC.

In 2018 Koppen et al. conducted a systematic review on the epidemiology of FC in children according to the pediatric Rome III and Rome IV criteria and reported a global prevalence of 9.5% according to the Rome III criteria [10]. No epidemiological studies using the Rome IV criteria were included in this systematic analysis.

These results were in line with a previous systematic review from Mugie et al. including 17 pediatric studies on FC prevalence, which was reported ranging between 0.7% and 29.6%, with a median of 12%. However, the authors included studies that considered different FC diagnostic criteria, varying from self-report to the Rome II and III criteria [11]. Similar global percentage of FC (14.4%) was found evaluating only studies conducted using the most recent Rome IV criteria [12]. To confirm this, Russo and colleagues in 2019 found no statistically significant difference comparing the prevalence of FC according to the Rome III and Rome IV criteria (17.3% vs. 18.2% *p* = 0.8) [13].

In 2023 Cenni et al. conducted a study on 740 Italian children aiming to evaluate the prevalence of FGIDs; FC resulted to be the most frequent disorder with a prevalence of 18.2% using Rome IV criteria, with a higher prevalence in children than in adolescents [14]. Another Italian study showed a similar prevalence of FC (16.1%) in children aged between 13 and 48 months [15]. Overall, FC can be defined as a common problem in children with up to 25% of visits to pediatric gastroenterologists and 3% of all general pediatric outpatient visits are due to FC [16].

## What is the etiology?

The etiology of FC remains unknown, but it seems to be multifactorial: genetic factors, lifestyle factors (e.g., diet and physical activity), and psychological disorders are likely to be involved. In particular, a crucial role seems to be played by the alteration of the rectal and pelvic floor functions through the brain-gut axis [17].

The main pathophysiological mechanism, especially in toddlers and young children, is the stool retention behavior. Retention of stool in the rectum causes water to be absorbed by the rectal mucosa resulting in increasingly lumpy and hard stools. This process leads to a vicious cycle of difficult defecation. Furthermore, if large stools are retained in the rectum, the rectal wall can become more and more distended resulting in fecal overflow incontinence, loss of rectal sensation, and loss of the normal urge to defecate [18, 19]. Stool retention behavior could be the consequence of previous painful or hard stool, which is worsened by an anal fissures history, or anxiety of toileting due to unpleasant toilets outside, or voluntary withholding due to lack of interest [1]. Changes in routine or diet, weaning, stressful events, entering kindergarten, intercurrent illnesses and even school can contribute to the problem [16]. As for socio-demographic factors, the data regarding the association between sex and constipation are conflicting. Notably, some studies found a slightly higher prevalence of constipation in girls [20, 21], while others reported similar prevalence rates between boys and girls [22]. A meta-analysis of 2018 found no association between gender and FC risk, as well as for age [10]. Geographic location seems to be associated with the prevalence of FC, with the greatest rates reported in the Americas and Europe compared to Asia [10, 11]. The higher prevalence in western countries suggests that cultural differences (e.g., lifestyle, environment, type of toilet) might play a role in FC pathophysiology. The difference in prevalence of constipation between the East and the West may be due to the greater amount of fiber in the Asian diet than most Western diets and to the different defecation posture. The squatting position is a common posture, particularly in asiatic rural areas, whilst most of the Western population defecate on a sitting toilet, changing the rectoanal angle [23]. However, it could be also due to the lack of epidemiological studies from other parts of the world [24].

It is commonly believed that a low-fiber diet contributes to constipation. Actually, the literature on this issue is still conflicting [25]. Although several pediatric studies observed an association between a lower intake of fiber and the development of constipation [26–28], other studies failed to demonstrate this association [29]. De Carvalho et al. showed no relation between low dietary fiber intake and constipation [30]. Similarly, in a study conducted on Japanese children dietary fiber intake resulted lower in the FC group compared to the non-FC group, but, binomial logistic regression analysis showed no correlation between intake of fiber and FC [31]. However, most of the non-constipated children do not consume the recommended amount of fiber [24].

Early child nutrition is also involved in FC: breastmilk has been reported to be a protective factor [15, 21, 32]. De Oliveira et al. confirmed a potential association between infant milk-type and childhood constipation concluding that exclusive breastfeeding in the first 6 months of age is a protective factor for constipation, with early effects on stool consistency, and later in life through epigenetic mechanisms and/or behavioral pathways [33].

Recent evidence demonstrates that FC may be the result of alterations of intestinal microbiota and microbiota-gut-brain axis. In line with the gut-brain axis theory, negative psychosocial factors (e.g., stressful life events, low quality of life) seem to be associated with a greater FC prevalence [10]. The composition of gut microbiota in infants depends on delivery mode [34] and cesarean delivery was associated with high risk of FC [35–37]. Finally, familiarity is another confirmed risk factor [38–40]. As a matter of fact, in 2010 Ostwani et al. published the results of a family study of children with and without functional constipation. First-degree relatives of the probands had a significantly higher rate of constipation than those in the control group (30% vs. 7% for siblings and 42% vs. 9% for parents; *p* = 0.009 and *p* < 0.001 respectively) [41]. Twin studies demonstrated that 59% of childhood constipation can be explained by genetic predisposition [29], although no gene mutations specifically associated with constipation have yet been found.

## Which are the signs and symptoms of functional constipation?

Symptoms of FC include hard, infrequent bowel movements, often accompanied by bloating and abdominal pain [42]. FC was the cause of acute abdominal pain in 50% of children who presented for a primary care visit and should be considered in this context [43].

Fecal incontinence, defined as the involuntary loss of stools in the underwear after being toilet-trained, is even frequently reported in children with FC. This is due to the overflow of soft stools passing around a solid fecal mass in the rectum. Children with FC had higher prevalence rates for fecal incontinence than children without constipation [44, 45]. An American study conducted on children aged 4–7 years reported a prevalence of fecal incontinence of 4.4%, of which 95% had FC [44].

The rectum and urinary tract are anatomically close and share innervation; this could explain the impact of anomalies in one system on the other [46]. Therefore children with FC often have also urinary tract disorders, such as incontinence and urinary tract infections [47]. The association between chronic FC and acute urinary retention could also be related to the chronically dilated rectum resulting in irritation of the vesical trigone, invagination of the posterior wall of the bladder, and urethral obstruction [48]. Loening-Baucke studied the effect of FC treatment on urological symptoms. Effective treatment of constipation led to the disappearance of daytime urinary incontinence, nighttime urinary incontinence, and recurring urinary tract infections, respectively, in 89%, 63%, and 100% of patients who did not have anatomical urinary tract abnormalities [49]. Thus, evaluation of bowel habits is recommended in the initial assessment of a child presenting with lower urinary tract symptoms [50]. A 2018 systematic review evaluating the prevalence of bladder symptoms in children with FC reported that lower urinary tract symptoms had a prevalence ranging between 37% and 64%; in particular, the prevalence of urinary tract infection ranged between 6% and 53% [51].

## What is the differential diagnosis?

The diagnosis of FC is exclusively clinical and does not require laboratory and/or radiological investigations. Through history and physical examination, alarm signs and symptoms that may suggest organic diseases can be identified and consequently further investigations can be required (Table 2). Indeed, current evidence does not support the use of abdominal radiography, colonic transit studies and rectal ultrasound to diagnose FC [52].

**Table 2:** Alarm signs and symptoms

- Delayed meconium passage
- Onset of symptoms <1 month
- Starting in neonatal period
- Ribbon stools
- Absent anal/cremasteric reflex
- Blood in the stools in the absence of anal fissures
- Failure to thrive or weight loss
- Abdominal distension
- Bilious vomiting
- Abnormal position of anus
- Tuft of hair on spine
- Deep sacral dimple
- Alteration lower extremity strength/tone/reflex

Even digital rectal examination should not be performed routinely, but only when children present a history of delayed meconium passage after birth, intractable constipation, or if Rome IV criteria are not completely fulfilled, in order to exclude an organic cause [52].

Physical examination should specifically focus on growth parameters, abdominal examination (muscle tone, distension, fecal mass), inspection of the perianal region (anal position, erythema, skin tags, anal fissures), and examination of the lumbosacral region (tuft of hair, deep sacral dimple, gluteal cleft deviation, flat buttocks). Alterations of lumbosacral region associated to a history of lower extremity weakness or loss of bladder continence or absent lower spinal reflexes (anal wink, cremasteric reflex and lower extremity deep tendon reflexes) raise concerns regarding a neurologic cause. In these cases, the magnetic resonance imaging should be taken into consideration. Currently, no evidence supports the use of MRI of the spine in children with chronic intractable constipation without other neurologic abnormalities [52].

It is important to inquire the age of symptoms beginning, the time of meconium emission, stools characteristics and the possible association of gastrointestinal symptoms, such as bilious vomiting and severe abdominal distension. In fact, symptoms onset in the first month of life and the delayed passage of meconium for 48 h in a full-term newborn should raise the suspicion of the presence of an organic condition such as Hirschsprung’s disease (HD). Therefore, in these patients a rectal suction biopsy is indicated [53]. However, there is another form called ultrashort Hirschsprung’s disease characterized by a very short segment of aganglionosis extending 2 to 4 cm proximal to the internal anal sphincter, with presence of ganglion cells on rectal biopsy. The clinical picture is similar to the classical short-segment HD (which involves most or all of the rectum and part of the sigmoid colon), except that the degree of constipation may be less severe and the complications of growth retardation and enterocolitis are less likely to develop. Differently, form short-segment HD, the diagnosis of ultrashort HD does not rely on rectal biopsy, since ganglion cells are normally identifiable. Therefore, the diagnosis is merely based on the demonstration of the failure of the internal sphincter to relax on rectal manometry [54, 55]. Cystic fibrosis also needs to be excluded in a newborn with delayed passage of meconium. As a matter of fact, the prevalence of constipation in cystic fibrosis patients is quite high, ranging widely from 10 to 57% [56].

Laboratory tests including thyroid hormones, calcium and serology for celiac disease should only be recommended in case of FC unresponsive to conventional therapy.

Chogle et al. in 2013 conducted a retrospective study on 7472 children with FC to study the prevalence of celiac disease, hypothyroidism and hypercalcemia. Only in a small percentage of children with constipation undergoing laboratory evaluation, an organic disease was diagnosed [celiac disease (1.7%); hypothyroidism (0.6%)] [57]. Similarly, Bennet et al. found that only a small proportion of their cohort (0.2%) had constipation as isolated symptom of hypothyroidism while patients evaluated for constipation with slow growth or slow growth alone were much more likely to be hypothyroid (2.5% and 2.2%, respectively) [58]. Regarding the correlation between celiac disease and constipation, in a multicenter study conducted on 2035 children with FC only 0.5% were diagnosed with celiac disease [59]. These results are in line with previous studies conducted in American [57] and Iranian [60] patients and with the latest guidelines on the management of constipation in children issued by ESPGHAN and NASPGHAN that recommend against systematic testing for celiac disease [52].

In order to differentiate between fecal incontinence due to constipation or to non-retentive fecal incontinence (NFI), total colonic transit time (CTT) using radiopaque markers can be used. Total CTT is significantly prolonged in constipated children compared with children with NFI. Such a differential diagnosis is essential, since the therapeutic approach is different: NFI children are best treated with a rigorous toilet training program and should not be treated with oral laxatives as they increase fecal incontinence.

Constipation has been reported in 4.6% of infants with cow’s milk allergy (CMA); the prevalence of food allergy underlying chronic constipation in children resistant to conventional treatment and presenting to tertiary clinics ranges between 28% and 78% [61]. However, no allergic tests, radiological or motility investigations achieve sufficient sensitivity and specificity to screen children for CMA-related constipation. A 4-week cows’ milk protein elimination diet may be considered for children with FC resistant to conventional treatment without alarm sign/symptoms of organic diseases [52].

Additionally, in the first 9 months of life, constipation must be differentiated from another FGIDs, infant dyschezia. Infants affected by this disorder present at least 10 min of straining and crying before successful or unsuccessful passage of soft stools, without any other health problems [3]. The stools are usually evacuated daily and are soft; infant dyschezia is thought to be due to a muscle coordination problem [3]. While dyschezia is a benign self-limiting disorder and no intervention is needed and manipulation for defecation should be avoided, in contrast FC requires an early treatment. In most infants, the symptoms begin in the first months of life, and resolve spontaneously in the majority of children after 3 − 4 weeks.

## What pharmacological treatments are recommended for disimpaction?

Fecal disimpaction represents the first step in pharmacological treatment being indicated to remove any hard fecal masses identified in the rectum. Based on the Rome IV criteria, two or fewer defecations per week for at least 1 month are required to start the treatment [3, 4].

Disimpaction can be accomplished with different osmotic and stimulant laxatives, rectal medications or a combination therapy. Among laxatives, according to the ESPGHAN/ NASPGHAN guideline, polyethylene glycol (PEG) is recommended as a first choice for disimpaction, due to the extensively reported high efficacy, good safety profile and tolerance [62]. Several prospective randomized studies compared oral PEG therapy to rectal enema for fecal disimpaction in children. Bekkali et al. found that the compared treatments with enemas once daily and PEG for six consecutive days was equally effective in 90 children with rectal fecal impaction [63]. A few years later, Miller et al. described a significant relief of symptoms on day 1 among 40 children treated with enemas compared to 39 children with oral PEG, while no difference was observed on day 5 between two groups. However, half of the children in the enema group reported to be ‘‘upset” with therapy, whereas no one in the PEG group [64]. Recently, a randomized controlled non-inferiority trial conducted by Strisciuglio et al. showed the short-term efficacy and safety of promelaxin microenemas compared to oral PEG in 158 infants and toddlers with FC [65]. However, since treatment with rectal enemas seems to be more invasive than oral PEG, the ESPGHAN/NASPGHAN guideline recommends the use of enemas once per day for 3 to 6 days, when PEG is not available [52]. Recommended dose for fecal disimpaction with PEG is 1-1.5 g/kg/day for a maximum of six consecutive days [52] (Table 3). In a prospective, double-blind, randomized study, Youssef et al found that doses of 1.0 and 1.5 g/kg given for 3 days in children with evidence of fecal impaction were more effective than lower doses, regardless duration of symptoms and severity of constipation [66]. High-dose PEG, however, is associated with a higher frequency of fecal incontinence during this treatment phase [62]. Therefore, the family should be prepared for potential worsening of overflow soiling in the first phase of the treatment. Various formulations of PEG have been developed, using PEG 3350 and PEG 4000 (with a molecular weight of 3350 and 4000 g/mol, respectively) with or without the addition of electrolytes. PEG 3350 and PEG 4000 are effective in childhood constipation without differences in efficacy between both treatments [67]. However, the addition of electrolytes has been demonstrated to deteriorate the taste and palatability of PEG with consequent poor adherence to the treatment [68, 69]. With regards to safety, as reported in a randomized double-blind multicenter study, children aged from 6 months to 16 years did not present any difference in the long-term use of PEG 3350 with electrolytes compared to PEG 4000 without electrolytes [70]. However, as described by Boles et al. in a retrospective observational study, PEG without electrolytes is associated with fewer side effects compared to PEG and electrolytes. Adverse events were reported in 11 out of 23 children treated with PEG and electrolytes compared with 1 out of 28 children receiving PEG without electrolytes. Side effects included electrolytes’ abnormalities, abdominal pain, nausea and vomiting [71].

**Table 3:** Pharmacological treatment in children with functional constipation

Oral Laxatives	Dosages
**Osmotic Laxatives**	
PEG 3350/4000 WHITE	All age groups: Maintenance: 0.2–0.8 g/kg/day in 1–2 doses Fecal disimpaction: 1–1.5 g/kg/day (maximum 6 days)
Lactulose	All age groups: 1–2 g/kg/day, in 1–2 doses
Lactitol WHITE	1–6 years: 0.5–1 g/kg/day in 2–3 doses 6–12 years: 10–30 g/day in 2–3 doses 12–18 years: 20–60 g/day in 2–3 doses
Magnesium hydroxide	2–5 years: 0.4–1.2 g/day, in 1 or more doses 6–11 years: 1.2–2.4 g/day, in 1 or more doses 12–18 years: 2.4–4.8 g/day, in 1 or more doses
**Stimulant Laxatives WHITE**	
Bisacodyl	3–10 years: 5 mg/day, in 1 dose/day (at night) 10–11 years: 5–10 mg/day, in 1 dose/day (at night) 12–18 years: 5–15 mg/day, in 1 dose/day (at night)
Senna WHITE	2–6 years: 2.5–5 mg, 1 or 2 doses/day 6–11 years: 7.5–10 mg, 1 dose/day 12–18 years: 15–20 mg, 1 dose/day
Sodium picosulfate	1 month to 4 years: 2.5–10 mg/day, in 1 dose/day 4–18 years: 2.5–20 mg/day, in 1 dose/day
**Rectal Laxatives DARK BLUE**	**Dosages**
Bisacodyl	2–10 years: 5 mg/day, in 1 dose/day (at night) > 10 years: 5–10 mg/day, in 1 dose/day (at night)
Sodium lauryl sulfoacetate WHITE	1 month to 1 year: 2.5 mL/dose (= 0.5 enema) 1–18 years: 5 mL/dose (= 1 enema)
Sodium docusate	< 6 years: 60 mL > 6 years: 120 mL
Sodium phosphate	1–18 y: 2.5 mL/kg, max 133 mL/dose
**Oral and rectal laxatives DARK BLUE**	**Dosages**
Mineral oil/liquid paraffin WHITE	2–11 years: 30–60 mL, in 1 dose/day > 11 years: 60–150 mL, in 1 dose/day
	3–18 years: 1–3 mL/kg/day, 1 or more doses/day (maximum 90 mL/day)

*Adapted from* [51]

However, the compliance to the treatment seems to be higher in children treated with PEG without electrolytes due to the better taste of the medication. Indeed, in a 4-week-trial conducted by Savino et al., macrogol without electrolytes was associated with a significantly lower proportion of patients reporting nausea when compared to macrogol with electrolytes [72]. To further improve the compliance and the safety, a new formulation without electrolytes, flavorings and excipients based on PEG 3350 treated by steam purification to remove impurities (ethylen oxide max 0.1 ppm and dioxane max 0.3 ppm) has been developed (Makropur®, trademark registered by SIIT). However, up to now, there are no published studies on this new formulation.

Other oral pharmacological options (e.g. lactulose, magnesium citrate, sodium picosulfate) can be considered for disimpaction, if high-dose oral PEG and enemas are not tolerated or ineffective [73]. Two different studies on children with different degree of constipation evaluated a disimpaction protocol combining the highest dosage used for PEG ranged from four to eight sachets per day (13–14.7 g/sachet) and the highest sodium picosulfate dose ranged from 7.5 mg to 10 mg per day. All patients were successfully disimpacted in the first study involving 44 children with FC, while in the subsequent study recruiting children with more severe constipation, the combined treatment was effective in removing the fecaloma only in half of the children [73, 74]. In children who fail first line disimpaction, stimulant laxatives such as senna and bisacodyl can be administered in combination with PEG [75]. Fecal impaction in the rectum unresponsive to oral medications or enema might require digital fragmentation and mechanical removal. Manual stool removal can be done under general anaesthesia, but structural injury to the anal sphincter after manual disimpaction may occur and contribute to sphincter weakness later in life [76].

## What pharmacological treatments are recommended for maintenance?

After successful disimpaction, it is necessary to continue with maintenance treatment to prevent the reaccumulation of feces and to keep the child, symptom free with regular soft bowel actions [77]. The ESPGHAN/NASPGHAN guidelines recommend the use of PEG as a first choice for maintenance treatment in all age groups, with the recommended dose of 0.2–0.8 g/kg/day and a starting dose of 0.4 g/kg/day, once daily or divided in more doses [52]. Dosages and dosing frequency should be individualized according to the clinical response. In details, treatment dosing need to be continuously adjusted on the basis of the consistency rather than the stool frequency, slightly increasing or decreasing the dose until symptoms remission. In a systematic review, Rachel et al. evaluated the safety and effectiveness of PEG in children aged younger than 24 months [78]. Based on limited studies [22, 79–81], this review demonstrated promising results regarding PEG safety. However, evidence to establish appropriate dosage regimens of PEG in children younger than 24 months was not available [78]. Therefore, if PEG is not available, lactulose is recommended and considered to be safe for all ages [52]. When osmotic laxatives alone are not sufficient, stimulant laxatives or lubricants (mineral oil) can be applied as an additional or second-line treatment of FC. The stimulant laxatives mainly used in pediatric clinical practice are sennosides, bisacodyl and sodium picosulfate. Although controlled trials on the use of these agents for constipation in children are still lacking [82], extensive clinical experience and expert opinion-based guidelines support their use as second-line treatment options. Due to the paucity of well-designed data in children on the efficacy and tolerability of the long-term use, children refractory to PEG therapy should be referred for the treatment to a specialized unit of pediatric GI [52] Table 3 shows the recommended dosages of most frequently used oral and rectal laxatives. Several pharmacological agents are currently being investigated as further options for childhood FC, including prucalopride, lubiprostone, linaclotide and pyridostigmine [74–79].

## How long the maintenance therapy should be continued?

The goal of the maintenance therapy is to keep stools soft and make defecations less painful and less frightening. Therefore, ESPGHAN/NASPGHAN guideline recommends assessing the efficacy after 2 weeks of treatment, in order to intensify it if necessary, and continuing the treatment for at least 2 months [52, 62]. Children who are in the developmental stage of toilet training should continue medication until toilet training is achieved [52, 83]. However, no randomized controlled trials (RCTs) evaluated the optimal duration for maintenance treatment [52]. However, even if the constipation lasts more than a year, maintenance therapy needs to be continued without any risk for the child [22, 62, 84]. Taking into account that a considerable proportion of children will need long-term PEG treatment in order to maintain clinical remission, the taste and palatability of orally administered drugs should be carefully evaluated to improve the adherence [69].

After a child has been treated for at least 2 months and regular defecation pattern is established, maintenance treatment should be gradually weaned rather than abruptly discontinued in order to prevent a relapse [62]. It is also recommended to evaluate symptoms again 2 months after the cessation of treatment, in order to prevent or detect relapses. After an adequate treatment, if the constipation reoccurs, it is mandatory to restart therapy at the optimal dosage. Therefore, it is important to carefully warn caregivers and children about this risk [6].

## Are there useful non-pharmacological therapies?

Normal fiber (i.e. 5 g + the age in years of the child) and fluid intake and normal physical activity in combination with education and demystification is recommended as first step in the treatment of FC [52]. Guidance for toilet training is added to the treatment for children with a developmental age of at least 4 years [52, 83]. Two systematic reviews based on 1 study conducted by Young et al. concluded that increasing oral fluid intake was not effective on constipation symptoms [85–87]. They also found limited clinical value of fiber in the management of FC. Increasing dietary fiber intake accompanied by extensive behavioural interventions did not increase bowel frequency or reduce the requirement for laxatives [88]. Other non-pharmacological treatment options such as prebiotics and probiotics, symbiotics, biofeedback, massage therapy, and alternative medicine have not shown to significantly improve defecation frequency [89]. A recent Cochrane review included 14 studies investigating the role of probiotics and concluded that there is insufficient evidence to recommend probiotics in successfully treating or changing the frequency of defecation [90].

In a recent systematic review and meta-analysis, Wegh et al. evaluated a total of 52 RCTs and showed that a cow’s milk exclusion diet, abdominal electrical stimulation and Cassia Fistula emulsion may be effective for increasing defecation frequency [89]. However stronger evidence is needed to confirm the efficacy of non-pharmacologic interventions for children with FC.

## What is the long-term prognosis?

The prognosis of FC in children was evaluated in a systematic review, including 14 heterogeneous prospective follow-up studies with a total of 1752 children [91]. This review reported that half of the children treated for FC were recovered and taken off laxatives after 6–12 months of follow-up. Approximately an additional 10% were symptom free while taking laxatives. After a follow-up of 1–2 years and 5–10 years, the recovery rate was 58% and 56%, respectively [91]. Children treated by pediatric gastroenterologists showed a higher recovery rate than children treated by general pediatrician [91]. Indeed, Borowitz et al. [92] reported that primary care physicians tend to undertreat childhood constipation. This is in line with the results of Bongers et al. [93] who demonstrated that a significant delay in treatment, defined as time between age at onset and first presentation at the department of pediatric gastroenterology, was negatively related to symptoms’ remission. However, in the long-term follow-up, several studies showed that a sizable group remains symptomatic regardless of treatment and can remain symptomatic into adolescence or adulthood [91, 94, 95].

## Conclusions

FC is a very common problem in childhood and occurs world-wide. Diagnosis is based on the Rome IV criteria after evaluation of a thorough clinical history and physical examination. Additional diagnostic testing is only indicated when an organic cause is suspected or if children do not respond to treatment despite optimal treatment.

The first step in treating FC involves education, demystification, lifestyle advice, and toilet training (when developmental age is at least 4 years). Pharmacological treatment with laxatives consists of three steps: disimpaction, maintenance treatment, and ultimately weaning if possible. PEG is still considered the first choice of laxative for both disimpaction and maintenance treatment. A large proportion of children remains symptomatic after 6–12 months of treatment; therefore, a strict follow-up is highly recommended.

## Data Availability

Not applicable.

## Abbreviations

CMA: Cow’s milk allergy
CTT: Total colonic transit time
FC: Functional Constipation
FGIDs: Functional Gastrointestinal Disorders
NFI: Non retentive fecal incontinence (NFI)
PEG: Polyethylene glicole
RCT: Randomized controlled trials
